# Exosomes, a New Star for Targeted Delivery

**DOI:** 10.3389/fcell.2021.751079

**Published:** 2021-10-08

**Authors:** Huizhi Chen, Liyan Wang, Xinling Zeng, Herbert Schwarz, Himansu Sekhar Nanda, Xinsheng Peng, Yubin Zhou

**Affiliations:** ^1^School of Pharmacy, Guangdong Medical University, Dongguan, China; ^2^Key Laboratory of Chinese Medicinal Resource From Lingnan, Ministry of Education, Guangzhou University of Chinese Medicine, Guangzhou, China; ^3^Department of Physiology, Yong Loo Lin School of Medicine, National University of Singapore, Singapore, Singapore; ^4^Biomedical Engineering and Technology Laboratory, Department of Mechanical Engineering, PDPM-Indian Institute of Information Technology, Design and Manufacturing, Jabalpur, India; ^5^Marine Biomedical Research Institute of Guangdong Zhanjiang, Zhanjiang, China

**Keywords:** exosomes, extracellular vesicles, exosome engineering, targeted delivery, drug delivery, gene delivery

## Abstract

Exosomes are cell-secreted nanoparticles (generally with a size of 30–150 nm) bearing numerous biological molecules including nucleic acids, proteins and lipids, which are thought to play important roles in intercellular communication. As carriers, exosomes hold promise as advanced platforms for targeted drug/gene delivery, owing to their unique properties, such as innate stability, low immunogenicity and excellent tissue/cell penetration capacity. However, their practical applications can be limited due to insufficient targeting ability or low efficacy in some cases. In order to overcome these existing challenges, various approaches have been applied to engineer cell-derived exosomes for a higher selectivity and effectiveness. This review presents the state-of-the-art designs and applications of advanced exosome-based systems for targeted cargo delivery. By discussing experts’ opinions, we hope this review will inspire the researchers in this field to develop more practical exosomal delivery systems for clinical applications.

## Introduction

Nanomaterials have provided new insights to carry biofunctional compounds for different biological applications, such as diagnosis, imaging, as well as therapies. For example, Liposomes have been reported for various passive and active targeted cargo delivery, which provide inspiration on the development of exosome-based targeted delivery system (Riaz et al., 2018). Exosomes are naturally derived nanocarriers which encapsulate various molecules in an enclosed phospholipid-bilayer membrane, acting as intercellular messengers to mediate both physiological and pathological processes (Thery et al., 2002). For instance, the development of cancer can be related to the intercellular transfer of proteins carried by the exosomes (Beckler et al., 2013). Exosomes are secreted by a variety of cells, and known to bear important information in the form of signaling molecules originated from parent cells, making them potential biomarkers for the early detection of cancers and other aging-related disorders, such as Alzheimer’s disease (Zhou et al., 2018; Jalalian et al., 2019). More importantly, natural exosomes transmit the messages to recipient cells through several mechanisms, including surface receptor interaction, membrane fusion, and receptor-mediated endocytosis, phagocytosis, and/or micropinocytosis. By utilizing these mechanisms, exosomes are able to deliver pharmaceutical compounds and bioactive ingredients to specific tissues and cells. Owing to their properties of natural generation, nano-scaled size, low immunogenicity, and the ability to cross several biological barriers, exosomes have been considered as promising drug carriers for targeted therapies. However, the therapeutic potential of exosomal delivery system is largely restricted by their uncertain loading efficiency, rapid clearance from systemic circulation, and sometimes weak targeting capacity. For cargo delivery, multiple approaches have been explored for exosomal loading of various drugs and other bioactive compounds of interest. Furthermore, various modifications have been applied to exosomes for prolonged blood circulation and higher tissue selectivity. These efforts make exosomes a new star for targeted delivery.

This review presents a comprehensive introduction on exosome-based delivery systems for targeted therapies. Firstly, the discussion on the biogenesis, composition, functions and appropriate sources of natural exosomes is presented to provide an understanding of therapeutic benefits and features of exosome-related tools. Additionally, the usage of exosome as carriers in therapeutic applications is reviewed according to various loading cargoes specially on genetic substances. In particular, we summarize the currently available methodologies for exosomal cargo loading and the state-of-the-art bioengineering strategies of membrane modification to increase the circulation time and targeting efficiency, which are highly significant in the development of delivery platform. Finally, we shed light on the challenges and potential solutions of exosome-based loading system, as well as the updates on current status of clinical trials. The present review is expected to bring significant value to advance the superior design of nano-complexes consisting of exosomes and therapeutic compounds on their promising pathways in the field of targeted delivery.

## Biogenesis, Compositions, and Functions of Exosomes

### Exosome Biogenesis

Extracellular vesicles (EVs) are cell-derived microvesicles basically composed of lipids, which bear biologically active cargoes such as proteins and nucleic acids to achieve the intercellular communications. According to the origin, formation and size, EVs are generally categorized into three groups, including exosomes (30–150 nm), plasma-membrane-budded microvesicles (50–1000 nm), and apoptotic bodies (1000–5000 nm) (Raposo and Stoorvogel, 2013; Yanez-Mo et al., 2015). It has been reported that exosomes are secreted by a wide range of cell types, which can be isolated from cell culture supernatants or various extracellular fluids, such as urine, blood and cerebrospinal fluid.

**FIGURE 1:**
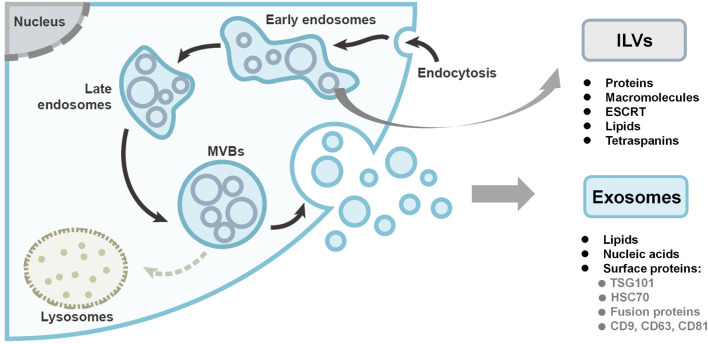
The biogenesis and compositions of exosomes. Exosomes originate as intraluminal vesicles (ILVs) through the inward budding of the plasma membrane within early endosomes. Early endosomes are then matured into late endosomes and ultimately multivesicular bodies (MVBs). Finally, the MVBs either undergo degradation or fuse with the plasma membrane to secrete the ILVs as exosomes. Figure redrawn from Kalluri and LeBleu (2020).

The biogenesis of exosomes starts from the inward budding of the plasma membrane, forming intraluminal vesicles (ILVs) within early endosomes, as shown in Figure 1. In the endosomal system, the membrane-enclosed organelles harbor the small ILVs, while the proteins and macromolecules are deposited into ILVs with the endosomal sorting complex required for transport (ESCRT) machinery, lipids (such as ceramide), and the tetraspanins (Hessvik and Llorente, 2018). Late endosomes are then matured into multivesicular bodies (MVBs). Finally, the fate of MVBs is to either undergo degradation by lysosomes or fuse with the plasma membrane to release the ILVs as exosomes (Thery et al., 2002; Kowal et al., 2014; Kalluri and LeBleu, 2020). In the biogenesis process, the ESCRT machinery is essential. Through RNAi screening in Hela cells, seven ESCRT proteins were found to affect exosome secretion (Colombo et al., 2013). Depletion of the ESCRT-0 and ESCRT-I proteins would decrease the production of exosomes, while knockdown of the ESCRT-III proteins would induce the exosome secretion. Chiu et al. (2016) used a time-lapse single-cell assay to monitor exosome secretion rate. It was demonstrated that each breast cancer cell releases 60–65 exosomes per hour while non-cancer cell has a 2.8-times higher exosome secretion rate, which is contradictory to other studies that relied on exosome isolation and measurement (Ventimiglia and Alonso, 2016).

### Exosome Compositions

Generally, exosomes encompass lipids, proteins, and nucleic acids. The exosome membrane mainly consists of lipid layers, containing cholesterol, sphingolipid, ceramide, and diacylglycerol, which differs from the membrane of other EVs (Doyle and Wang, 2019). The surface proteins of exosomes are dependent on their endosomal pathway, some of which can be used as exosomal markers, including tumor-sensitive gene 101 (TSG101), heat shock proteins (HSC70), fusion proteins (flotillin and annexin), tetraspanins (CD9, CD63, and CD81) (shown in Figure 1). Nucleic acids are also found in exosomes including double stranded DNA (dsDNA), messenger RNAs (mRNAs), and micro-RNA (miRNAs).

Notably, the protein and nucleic acid profiles of exosomes are diverse, which are strongly correlated to the cell types of the originating cells, as well as to the cell status like inflammation, viral infection, cancerous and neurodegenerative situation. For instance, exosome released from IL-1β-stimulated synovial fibroblasts could initiate osteoarthritis-changes in articular chondrocytes, and activate migration and tube formation of human umbilical vein endothelial cells (Kato et al., 2014). Besides, exosomes derived from stage I pancreatic ductal adenocarcinomas could trigger liver pre-metastatic niche formation. It may correlate to the higher level of macrophage migration inhibitory factor expressed in these exosomes, compared to that from the patients without metastatic progression (Costa-Silva et al., 2015). Based on these findings, exosomes isolated from biological fluids have been considered as promising biomarkers for early diagnosis and evaluation of disease conditions, in particularly, tumor progression and metastasis (Azmi et al., 2013; Zhu et al., 2021). More importantly, these distinct surface molecules are facilitating ligand-receptor binding, mediating specific organotropic accumulation. It has been reported that specific integrins on cancer exosome surface were able to determine organotropic metastasis (Hoshino et al., 2015). Exosome secreted from breast cancer cells metastasized and uptook by lung fibroblast and epithelial cells to prepare the pre-metastatic niche. In particular, the lung targeting properties was owing to exosome surface integrins α_6_β_1_ and α_6_β_4_, while integrin α_*V*_β_5_ of liver tumor exosome was correlated to liver metastasis. The specific surface integrins from parent cells have potentials for targeted guidance in delivery applications.

### Exosome Functions

To modulate physiological and pathological pathways, exosomes work as message transporters in intercellular communication *via* content transfer and interaction with the recipient cells. To date, exosomes have been widely reported to take part in physiological and homeostatic processes (Ventimiglia and Alonso, 2016; Baruah and Wary, 2020). Exosomes released from neural progenitor cells promoted neuronal differentiation to regulate neurogenesis *via* transfer of key miRNAs like miR-21a (Ma et al., 2019). In a study on a developing tooth organ, it was found that exosomes diffuse through the basement membrane, and mediate epithelial-mesenchymal crosstalk (Jiang et al., 2017). Epithelial exosomes interact with mesenchymal cells to induce dentin sialoprotein production, while mesenchymal exosomes activate epithelial cells to secrete basement components. The miR135a carried by epithelium-derived exosomes takes part in Wnt/β-catenin activation to induce dentin matrix production of the mesenchyme. Exosomes also function to modulate the disease development. Exosomes shed from gastric cancer cells activate the NF-κB signaling pathway in macrophages to increase the level of pro-inflammatory factors, resulting in the promotion of cancer progression (Wu et al., 2016). Adipocyte exosomes from obese donors regulate fibrotic signaling pathways, i.e., TGF-β and Wnt/β-catenin, to induce chronic inflammation (Ferrante et al., 2015). In summary, exosomes have demonstrated their capacity to manipulate important cell-cell communication in the form of cross talk *via* several signaling molecules to regulate both physiological and pathological processes, showing their potential applications for *in vivo* delivery.

Generally, two mechanisms are involved in the interaction between exosomes and target cells. In some cases, exosomes enter the target cells through micropinocytosis or endocytosis, or they fuse with the cellular membrane and release their cargo to activate various signal pathways. Alternatively, exosomes interact with the recipient cells through ligand-receptor binding on the surface to stimulate cascade responses. Whichever of the mechanisms involved, the specific binding or accumulation of exosomes endows their potential targeting capacity for delivery of an extensive range of molecules.

## Exosomes as Delivery System for Therapeutic Applications

Although the employment of exosomes as theragnostic tools for diseases has been reported, the attractive biological properties and functions of endogenous exosomes inspire further exploration in both the scientific and the clinical field. Considering their intrinsic characteristics, including low immunogenicity, innate stability, and the ability to cross biological barriers, the utility of exosomes as therapeutic vehicles for functional cargo delivery is favorable. Comparing exosomes to artificial liposomes, which share a similar lipid bilayer structure, biologically originated exosomes with enhanced loading capability of biological molecules are more efficient at entering recipient cells and delivering therapeutic agents upon administration by injection (Fuhrmann et al., 2015; Kamerkar et al., 2017; Song et al., 2021).

### Delivery of Small Molecules

**FIGURE 2:**
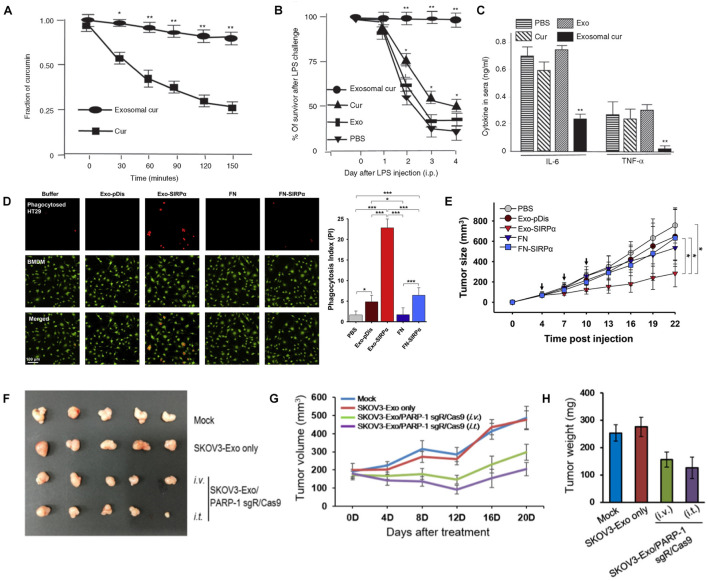
**(A–C)** Curcumin encapsulated in exosomes improved the bioavailability after intraperitoneal injection **(A)**; exosomal curcumin reduced lung inflammation **(B)** and increased survival **(C)** of lipopolysaccharide-induced septic shock mice. **(D,E)** Exosome containing SIRPα (Exo-SIRPα) enhanced phagocytosis of HT29 cells by bone marrow derived macrophages (BMDMs) **(D)** and *in vivo* antitumor efficacy **(E)**. **(F–H)** Exosomes loaded with PARP-1 sgR/Cas9 reduced tumor size **(F,G)** and weight **(H)** on SKOV3 xenograft mice after intravenous or intratumoral injection (*p < 0.05, **p < 0.01 and ***P < 0.0001). Figure reprinted from Sun et al. (2010); Kim et al. (2017), and Cho et al. (2018).

Various studies have shown that exosomes are prospective vehicles for therapeutic small molecules, such as curcumin, paclitaxel and doxorubicin (DOX). Sun et al. (2010) encapsulated curcumin into exosomes, and found curcumin to be much more concentrated in exosomes in the plasma than free curcumin (Figure 2A). Additionally, the exosome-encapsulated curcumin was able to reduce inflammation, resulting in higher survival of lipopolysaccharide-induced septic shock mice (Figures 2B,C). Kim et al. (2016) developed macrophage-derived exosomes loaded with the chemotherapeutic drug paclitaxel through sonication, which not only exhibited 50 times higher cytotoxicity to drug-resistant cancer cells, but also produced a stronger antineoplastic effect in mice with pulmonary metastases (compared to the control). Furthermore, the same group incorporated aminoethylanisamide-polyethylene glycol into paclitaxel-loaded exosomes, aiming to target the sigma receptor which is highly expressed in lung cancer cells (Kim et al., 2018). The modified exosome-based platform showed increased drug uptake and superior accumulation in cancer cells upon systemic administration with improved therapeutic indices. In a recent study, exosomes isolated from LIM1215 (a human colorectal cancer cell line) cells were loaded with doxorubicin and engineered with superparamagnetic iron oxide nanoparticles and the A33 antibody (Li et al., 2018), which demonstrated an excellent targeting ability to colon cancer with reduced heart cytotoxicity.

### Delivery of Proteins

Various studies investigated the exosomes-mediated delivery of large biomolecules like peptides and proteins such as enzymes, antigens, cytoskeletal proteins, and transmembrane proteins. To treat inflammatory and neurodegenerative disorders, Haney et al. (2015) developed an exosomal delivery platform for catalase, a potent antioxidant. The exosomes containing catalase could be delivered to Parkinson’s disease mouse brain upon intranasal administration and taken up by neuronal cells. Cho et al. (2018) loaded membrane protein signal regulatory protein α (SIRPα) into exosomes for antitumor treatment by blocking the CD47 receptor on tumor cells. In the study, they found that phagocytosis of tumor cells and inhibition of tumor growth were enhanced when compared with the case using ferritin nanocages as vehicles (Figures 2D,E). In another study, exosomes harboring PH20 hyaluronidase were developed to advance tumor penetration *via* hyaluronan degradation (Hong et al., 2018). More particularly, the exosome-based delivery of both PH20 hyaluronidase and DOX could effectively enhance tumor inhibition.

### Delivery of Genetic Substances

Capable of protecting nucleic acids from degradation, exosomes appear to be ideal carriers for nucleic acids (like siRNAs and miRNAs) in gene therapy. Momen-Heravi et al. (2014) employed B cell-derived exosomes for the delivery of miRNA-155 inhibitor to reduce lipopolysaccharide-stimulated TNFα production in macrophages. In another study, tumor suppressor miRNA miR-199a-3p was loaded into exosomes isolated from omental fibroblasts of ovarian cancer patients (Kobayashi et al., 2020). Both *in vitro* and *in vivo* studies demonstrated the miRNA-exosome effectively suppressed c-Met production and inhibited cancer cell proliferation and invasion. Based on the identification that exosomes from autologous breast cancer cells show effective lung targeting capability, Zhao L.W. et al. (2020) developed a new type of biomimetic nanoparticles consisting of albumin and siS100A4 with the coating of exosome membrane, and this exosome-based siRNA delivery system was capable of significant inhibition of postoperative breast cancer metastasis. Additionally, it was observed that the exosome-mediated delivery of siRNA or miRNA is more efficient and less cytotoxic compared to the conventional transfection methods (Momen-Heravi et al., 2014; Jin et al., 2016; Kobayashi et al., 2020; Zhao L.W. et al., 2020).

Recently, CRISPR/Cas9 genome editing technology has become attractive to scientists and engineers due to the unprecedented precision, efficiency and flexibility of changing, deleting, or replacing a specific genes (Ran et al., 2013). The development of CRISPR/Cas9 has markedly spurred the treatment for a broad range of diseases. However, current vehicles delivering CRISPR/Cas9 are mainly restricted by their immunogenicity. As natural carriers, exosomes exhibit great potential to encapsulate and deliver CRISPR/Cas9 system into the cells. Kim et al. loaded tumor-derived exosomes with CRISPR/Cas9 against poly (ADP-ribose) polymerase-1 (PAPR-1) to inhibit cancer cell propagation (Kim et al., 2017). The results showed that exosomes as carriers confer an efficient delivery of CRISPR/Cas9 plasmids with cancer cell tropism, and the loaded exosomes facilitated advanced anti-tumor effect *in vivo* (Figures 2F–H). Luo N.N. et al. (2021) reported that hepatic fibrosis could be attenuated by exosome-mediated CRISPR-dCas9-VP64 through activating NHF42 expression of hepatic stellate cells. Although intensive studies have been performed on the development of exosome-based delivery systems, their clinical applications are still limited by the low efficiency of loading giant molecules such as plasmids. To improve the encapsulating efficiency of Cas9 proteins, Ye et al. (2020) engineered exosomes through conjugating CD63 and Cas9 with green fluorescent protein (GFP) and GFP antibody respectively, and the affinity of the GFP antibody thereby assisted the capture and loading of Cas9 protein into the exosomes. The modified exosomes loaded with CRISPR-Cas9 components exhibited enhanced abrogation of the targeted gene. In Lin’s study, exosomes were hybridized with liposomes through simple incubation to increase the loading efficiency of CRISPR/Cas9 system, and it was observed that the resultant hybrid exosomes could be endocytosed by mesenchymal stem cells and express the encapsulated genes (Lin et al., 2018).

### Improvement on Targeting Ability

Exosomes hold great promise as nanocarriers for various treatments owing to their unique ability to cross biological barriers and to migrate into the tissue areas without blood supply. However, sometimes the insufficient targeting ability restricts their clinical applications, which requires advanced strategies for improved their efficacy. To enhance the tissue-specific targeting ability, Tian et al. (2018) conjugated curcumin-loaded exosomes with c(RGDyK) peptide through surface functionalization for the treatment of cerebral ischemia. The results from a mouse model showed the engineered exosomes could cross the blood-brain barrier (BBB) after intravenous administration and reach the lesion region of the ischemic brain. Liang et al. (2020) developed miR-140-loaded exosomes bearing the chondrocyte-affinity peptide for the treatment of osteoarthritis, and it was observed that the tagged exosomes upon intra-articular injection efficiently reached the deep cartilage regions across the dense extracellular matrix and inhibited osteoarthritis progression, suggesting a potential cell-free treatment method for osteoarthritis.

**FIGURE 3:**
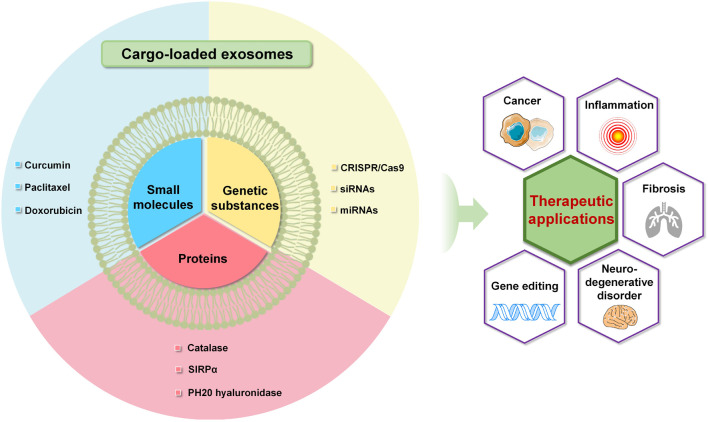
Advanced exosome-mediated delivery systems of small molecules, proteins and genetic substances toward various therapeutic applications.

Overall, exosomes as vehicles for various molecules have exhibited great potential for clinically therapeutic applications (Figure 3). In order to enhance the effectiveness of the treatment, exosomes need to be engineered to improve the loading efficiency and capability. Furthermore, biofunctional engineering endows the exosomal delivery with enhanced targeting capability and improved circulation time, facilitating the efficient delivery of cargoes to specific cells or tissues.

## Loading Approaches

**FIGURE 4:**
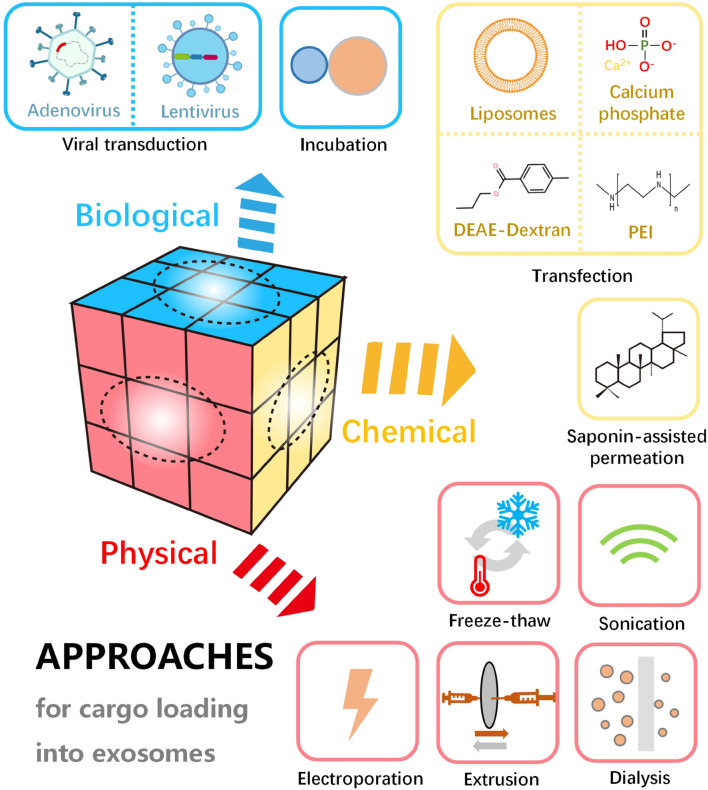
Schematic diagram of various approaches for cargo loading into or onto exosomes, including physical, chemical, and biological methods.

Loading of various active substances in/on exosomes endows them with specific biological functions. Efficient encapsulation strategies for exosomes are essential for favorable delivery of therapeutic cargo and designed targeting elements. To date, multiple approaches have been developed for the loading of a wide range of active molecules into the exosomes. According to the working mechanisms, these approaches can be divided into three main categories, including physical, chemical and biological methods (Figure 4). Physical loading is typical methods, including freeze-thaw, sonication, electroporation, extrusion, etc. As traditional loading methods, sonication and freeze-thaw are widely applied. In addition, physical energy in the form of optical, electric and mechanical interaction are sometimes employed to load the therapeutic molecules into the exosomes. However, the physical destruction often affects the function and integrity of the phospholipid membrane. Compared to the physical methods, loading with chemical agents is milder and more effective in some cases. For example, in chemical loading processes, saponin-assisted permeation and transfection are usually applied to facilitate molecule encapsulation through membrane permeabilization and electrostatic interaction, respectively. Further, biological strategies such as incubation and viral transduction are able to result in a high loading efficiency. Loading of active molecules (i.e., therapeutic or targeting molecules) can be carried out through simple incubation or complicated viral transduction based on the biological traits of the membrane and vector. With the viral transduction loading approaches, the alterations on exosomal structure and functionality may be minimized. It is noteworthy that efficient loading into exosomes can be achieved through various approaches, but the exosome and cargo may be damaged by the destructive engineering. Therefore, key parameters should be optimized for desirable loading, such as the power of physical interaction, operation time, cell type concentration of reagents and exosomes.

### Physical Methods

#### Sonication

Inspired by the preparation of liposomes, the sonication process has been applied to load cargo to exosomes. Probe sonication is frequently used to cause the formation of transient pores or even the breakdown and reformation (Kim et al., 2016) of naïve exosomes so that the cargoes, including nanoparticles (Jang et al., 2021), small chemical drugs (Lin et al., 2019; Haney et al., 2020), siRNA (Lamichhane et al., 2016), and proteins (Sancho-Albero et al., 2019), can be encapsulated in the vesicles by simple diffusion. The loading efficiency is reported to be relatively high (Sancho-Albero et al., 2019). Unexpectedly, the local enormous energy can lead to heating and damage of agents in some cases. Therefore, an optimized temperature control or other processing conditions (like pulse, power output) are essential. Interestingly, Haney et al. (2020) noticed that sonication of exosomes with Paclitaxel (PTX) with only the “off” period cooling resulted in more efficient drug loading, compared to ice cooling for the whole treatment, possibly owing to the slightly elevated temperature making the lipid bilayers less rigid. Moreover, they found that a pH closed to the (isoelectric point) pI resulted in significantly enhanced DOX encapsulation, accompanied with decreased charge and increased hydrophobicity of the drug-load exosomes. At the end of sonication, co-culture of the mixture at room temperature (RT) for 1 h can be utilized for reconstitution of the exosome membrane and subsequent improvement of stability (Salarpour et al., 2019; Wang P. et al., 2019). Remarkably, the loading molecules of interest may be attached to the outer layer of the exosome membrane (Kim et al., 2016; Sancho-Albero et al., 2019), and the size of the exosomes tends to be smaller compared to other physicochemical approaches (Luo L. et al., 2021). Moreover, the sonication treatment may remove the tumorigenic traits of tumor-derived exosomes by eliminating their original internal substances such as RNAs (Jang et al., 2021).

#### Electroporation

Electroporation, whose parameters can be well controlled in a simple way has become one of the most frequently used methods for loading various molecules in exosome. With the help of a conductive buffer, recoverable pores are opened at the moment of applying an external electric pulse, allowing the molecules to get across the membrane (Shigekawa and Dower, 1988). In the processing, the cargoes, such as miRNA, SPIONs, small molecule chemotherapeutics and bioactive macromolecules are mixed with the exosomes directly (instead of the parent cells), followed by the transfer to a chilled electroporation cuvette and voltage applied. The condition of the applied potential can vary greatly from 0.1 to 1000 kV (Kim et al., 2016; Asadirad et al., 2019), dependent on the origin and concentration of the exosomes. However, the aggregation of cargo like siRNA, proteins, DNA, has been a limitation for the electroporation method (Johnsen et al., 2016). The complex formation between metal ions from the electrodes and hydroxide ions from the conductive buffer may be responsible for the aggregation (Kooijmans et al., 2013). Thus, the involvement of disaccharide trehalose, citric acid and EDTA in the electroporation buffer, and optimization of electroporation parameters can contribute to the prevention of the nucleic acid aggregation and an overestimated encapsulation efficiency (Kooijmans et al., 2013; Hood et al., 2014; Johnsen et al., 2016).

#### Freeze-Thaw

The freeze-thaw method is a relatively mild process to load miRNA and proteins (Hajipour et al., 2021), involving the repeated fusion of lipid bilayer. Generally, the exosomes are rapidly frozen at below –70°C, followed by thawing at RT, for several cycles. Generally, a minimum of 3 cycles of thawing is required while 5∼10 cycles are preferred (Sato et al., 2016). Won Lee et al. (2020) has demonstrated that the freeze-thaw loading strategy of therapeutic agents has no significant impact on the structure of exosomes. The mean diameter could vary by only 7 nm after the loading procedure (Won Lee et al., 2020). However, in term of encapsulation efficiency, no obvious improvement was observed (though this is acceptable sometimes). Recently, in order to advance the loading efficiency, the freeze-thaw approach was modified by incorporating the incubation and sonication processes (Tran et al., 2019).

#### Extrusion

**FIGURE 5:**
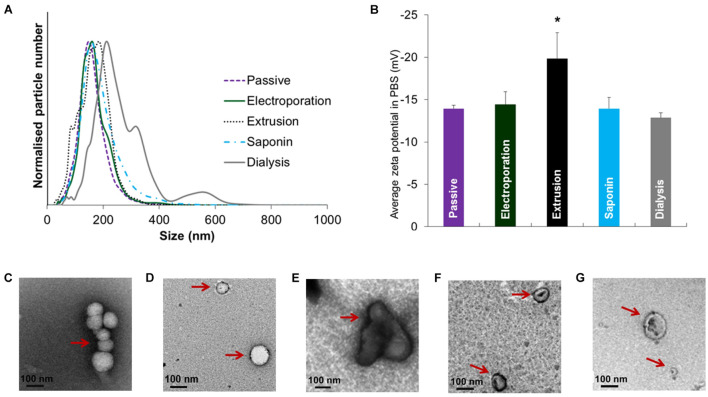
Size distribution, zeta potential and morphology of EVs after drug loading with various methods. **(A)** Size distribution and **(B)** zeta potential after loading of porBA into vesicles from MDAs. Transmission electron microscopy images of EVs from MDAs indicated that shape and size of EVs after **(C)** passive, **(D)** electroporation, and **(F)** saponin treatment are not altered (arrows). While **(E)** extrusion, and **(G)** hypotonic dialysis induced aggregate formation and broader size range of EVs, respectively (arrows) (*p < 0.05). Figure reprinted from Fuhrmann et al. (2015).

Like sonication methods, the employment of the extrusion technique in exosome research is derived from its application in liposome-based drug delivery. With an extruder equipped with a heating block, the mixture of exosomes and cargoes is pushed repeatedly through polycarbonate membranes with 100∼400 nm pores, during which the cargo can diffuse into the exosomes at a controlled temperature (Oskouie et al., 2019). Extrusion processing has demonstrated a relatively high packing efficiency with uniform size distribution (Haney et al., 2015). Nevertheless, intensive extrusion and excessive shear stress excessively may alter the properties of exosome membranes such as the variation in the zeta potential and the structure of surface proteins. Fuhrmann et al. (2015) reported that changes in zeta potential and cytotoxicity were observed after a 31-times repeat of packing porphyrins/exosomes through extrusion. Interestingly, the authors compared the effect of different loading methods on size distribution, zeta potential and morphology of EVs, and found a significant change in the extrusion treatment (Figure 5; Fuhrmann et al., 2015). In addition to the wrapping of drugs, this method has been extensively employed in the engineering of exosome-mimetics, such as the extrusion of donor cell membranes through filters with serially diminishing pore sizes.

#### Other Physical Methods

Based on optic-activated reversible protein-protein interaction, Yim et al. (2016) employed a light-induced strategy for the loading of therapeutic proteins into the exosomes. Under blue light (460 nm) illumination, CRY2-conjugated protein cargoes docked with exosome transiently, which were then introduced into the exosomes *via* the process of endogenous biogenesis. Finally, the cargo was deposited into the intraluminal space of the exosomes after the removal of illumination source. In addition, some reports suggest that the desired therapeutic molecules could be enveloped into exosome *via* hypotonic dialysis (Wei et al., 2019; Jeyaram et al., 2020). Dialysis is a passive loading method relying on osmosis, which does not involve the exposure of exosomes and cargoes to external force (Seyfizadeh et al., 2019). The molecular weight of the dialysis membranes is one of the most important factors affecting the dialysis outcome. Nowadays, this loading method is attractive due to the superiority in loading efficiency and the elimination of additional purification (Fu et al., 2020).

### Chemical Methods

#### Saponin-Assisted Permeation

Simple incubation with surfactants like saponin or triton can increase the membrane permeability of exosomes and facilitate cargo loading, among which saponin is the most widely used surfactant in this field (Huda et al., 2021). As a natural surfactant, saponin interacts with the membrane-bound cholesterol, which creates pores and increases the permeability of cell/exosome membranes (Jamur and Oliver, 2010). In the presence of 0.01∼0.2% saponin, small drug molecules such as porphyrins, DOX and PTX, and proteins including HGNs, β-glucuronidase, catalase were successfully incorporated into the exosomes (Fuhrmann et al., 2015, 2018; Haney et al., 2015; Sancho-Albero et al., 2019; Thakur et al., 2020), but the loading capacities were distinct at different processing conditions. For example, Fuhrmann et al. (2015) showed that a dramatical increase (up to 11 folds) of 4,4′,4″,4″′-(porphine-5,10,15,20-tetrayl) tetrakis(benzoic acid) (porBA) loading into MDA-MB231 (an epithelial, human breast cancer cell line) cell-derived exosomes could be achieved by treating the exosomes with 0.01% (w/v) saponin at RT for 10 min, in comparison to some other passive methods. For instance, Sancho-Albero et al. (2019) reported a relatively low encapsulation yield (16.4%) by mixing B16-F10-exosomes with HGNs and 0.2% saponin by stirring for 20 min at RT. Under proper conditions, the strategy did neither compromise the integrity of the vesicles nor affect the efficacy of the drug such as the catalytic activity of β-glucuronidase (Fuhrmann et al., 2018). However, due to the known hemolytic risk of saponin, its applications *in vivo* might be restricted in some situations, for instance, in the case of high saponin dose required for specific cargo loading or lack of extra purification process due to technical limitation (Sancho-Albero et al., 2019).

#### Transfection Reagent-Based Encapsulation

It is generally recognized that the macro-biomolecular nature of nucleic acids poses challenges in their *in vivo* delivery. The natural presence of DNA, RNA, miRNA and non-coding RNA in exosomes suggests their compatibility for nucleic acid-based drug delivery systems. The chemical transfection reagents, such as calcium phosphate (Zhang et al., 2017), diethylaminoethyl (DEAE)-Dextran (Lokossou et al., 2020), polyethylenimine (PEI) (Zhupanyn et al., 2020), and liposomes, are able to facilitate the nucleic acid loading on/into exosomes, of which liposomes are the most widely used reagents (Wang H. et al., 2021; Wang Q.Z. et al., 2021; Wang X. et al., 2021). The mechanism is the same as for regular transfection where calcium phosphate forms co-precipitates with nucleic acids, while other reagents usually form positively charged micro/nano vehicles for complexation of negatively charged nucleic acids *via* electrostatic interaction.

Calcium phosphate (Zhang et al., 2017) and liposomes (Lu et al., 2019) were conventionally used as transfection reagents for cells. Recently, they were reported to efficiently load DNA into exosomes. It is noted that a commercial reagent based on a cell-penetrating peptide has been developed for the direct loading of siRNAs and miRNAs into the exosomes (Aqil et al., 2019; Nordmeier et al., 2020; Wang et al., 2020). However, the reagent’s residuals may affect the vesicle reconstitution process and exosome functionality. Thus, the additional washing and purification steps should be included to avoid the adverse effects. Moreover, it is not easy to separate exosome vesicles from the involved reagents in practical operations, posing the difficulty to distinguish whether the exosomes or complexes of transfection agents exert the functional effect when investigating the exosomes (Wahlgren et al., 2012). Meanwhile, it is unknown whether all genetic cargoes has been loaded into the exosomes or is just adhering to the surface of the exosomes (Shtam et al., 2013). Finally, in a general transfection reagent-based procedure, target nucleic acids are firstly mixed with appropriate reagents to form complexes, followed by an incubation step with parent cells for initiation of transfection. The specific nucleic acids are then expressed as desired proteins or peptides that are packaged into the exosomes afterward (Fu et al., 2020) *via* the classical biogenesis pathways (Figure 1)—This can be utilized for loading specific macromolecules into exosomes, and will be detailed in the later section on exosome modification. Further, the different ways of complex formation may induce potential variations on the reproducibility or stability.

### Biological Methods

#### Incubation

Incubation seems to be the easiest and most direct loading method which is achieved by mixing the proposed molecules with parent cells or exosomes. With passive transport mechanism driven by a concentration gradient, therapeutic small molecules can pass through the membranes and then enter the target exosomes or cells (followed by a secretion of cargo-loaded exosomes) (Pignatello, 2013). Despite the low encapsulation efficiency, it is essentially the most basic method, as it can maintain the activity of cargoes and exosomes (such as size and morphology), for example, superior to sonication at these aspects (Haney et al., 2015; Brossa et al., 2021).

By tuning the cells/exosomes-to-drug ratio, the loading capacity of siRNA can range from 73 to 30000 per vesicle (Didiot et al., 2016; Stremersch et al., 2016). In addition to concentration manipulation, incubation temperature, volume and time also make a difference (O’Loughlin et al., 2017). It is well-known that the rise in temperature generally increases the fluidity of the lipid membranes, which may be beneficial for cargo loading. However, it should be noted that an inappropriate high temperature may pose the risk of protein denaturation. Besides, the balance between the electrostatic repulsion and space needs to be considered, which correlates to the volume used. To assess the influence of different incubation times, Giassafaki et al. (2021) mixed siRNA with the MRC-5- and Vero-exosomes *via* stirring the mixture at 500 rpm for 90 min, 4 and 17 h at RT, and found that 90 min of incubation resulted the best for loading the exosomes with chemically synthesized oligonucleotides.

#### Viral Transduction-Based Strategies

The viral transduction-based strategy is popular for genetic drugs for therapeutic applications, which has now been applied to exosome-based delivery systems, owing to their stable and definite transfection abilities (Sharma et al., 2018). After virus (i.e., lentivirus and adenovirus) infection, the donor cells overexpress specific genes or regulate transcription, and the gene or gene expression products are then loaded into exosomes or exosomal membranes during the secretion process (Antimisiaris et al., 2018). Viral transfection is considered to be an important strategy for stable loading of genetic-based cargoes into exosomes (Fu et al., 2020).

**FIGURE 6:**
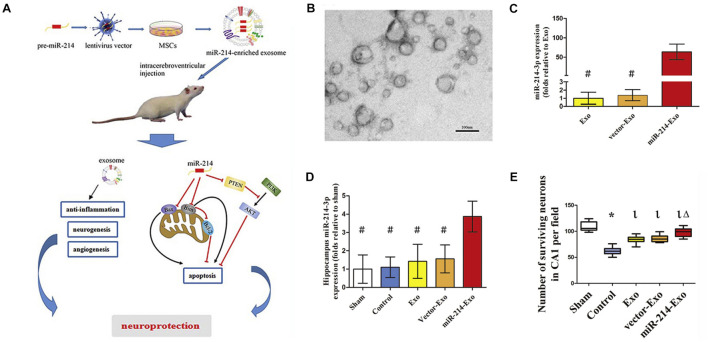
Gene-modified exosomes protect the brain against prolonged deep hypothermic circulatory arrest. **(A)** Schematic illustration of the principle. **(B)** Electron microscopic image of miR-214–enriched exosomes. **(C)** miR-214-3p expression was enhanced significantly in cultured MSCs transfected with premiR-214 (^#^*p* < 0.001 vs miR-214-Exo). **(D)** Intracerebroventricular injection of miR-214–enriched exosomes markedly enhanced the expression of miR-214-3p in the hippocampus (^#^*p* < 0.001 vs miR-214-Exo). **(E)** Number of surviving neurons in CA1 area of hippocampus post administration (*p < 0.001 vs sham group, ^l^*p* < 0.001 vs control group and ^Δ^*p* < 0.005 vs Exo group or vector-Exo group). Figure reprinted from Shi et al. (2021).

Viral transduction is suitable for a variety of cells to load them with high efficiency, which may solve the problem of chemical transfection that is inefficient for some cells. For example, Shi et al. (2021) successfully transduced (mesenchymal stem cells) MSCs with lentiviral vectors containing *pre-miR-214*, resulting in a significant enhancement of the *miR-214* production in MSCs-derived exosomes. As a result, the overexpress of *miR-214* enhanced the exosome-conducted neuroprotection against cerebral injury induced by deep hypothermic circulatory arrest (DHCA) (Figure 6; Shi et al., 2021). Yi et al. (2019) stably transduced the anti-apoptotic *miR-30b-3p* into MSCs with the assistance of lentivirus, and noticed the potential of MSC-exosomes bearing *miR-30b-3p* in preventing acute lung injury (ALI). Besides lentiviruses, adenoviruses are another commonly employed vector type for the generation of proposed cargoes in exosomes. With adenoviral vectors, Wang B. et al. (2019) could produce engineered exosomes from satellite cells containing *miR-26a* with muscle targeting ability.

Although viral transduction-base strategy may extensively enrich the exosome functions, the mechanism is still not fully revealed. In addition, the pathogenicity and teratogenicity of the viruses may be preserved and inherited in exosomes, resulting in safety risks (Zhao X. et al., 2020), and it is worth noting that laborious and time-consuming procedures are needed in this approach. Indeed, both viral transduction and the abovementioned chemical transfection methods are a popular way to load bio-macromolecules into exosomes, which will be further discussed in the later sections.

### Summary on Loading Approaches

Table 1 summarizes the abovementioned features of the loading methods. A number of approaches have been applied for cargo loading into exosomes. These approaches mainly include physical methods (sonication, electroporation, freeze-thaw, and extrusion), chemical methods (saponin-assisted permeation and transfection) and biological methods (incubation and viral transduction), while each method has its own advantages and drawbacks. However, a “perfect” addressing all the demands is still missing. For example, the powerful disturbance from physical methods poses the possibility to destroy the morphology or integrity of the exosomes, especially in electroporation. Freeze-thaw is considered to be a relative wild strategy, but it may result in an uncertain loading efficiency. On the other hand, biochemical approaches suffer some other issues, for instance, the biological methods are facing multiple challenges including low efficiency, long encapsulation cycle and uncontrollable loading process, while chemical strategies are struggling with extra complicated purification steps. Besides, saponin-assisted permeation (chemical approach) leads to certain concern owing to their potential hemolytic function, although the method can incorporate proteins into exosomes directly with high encapsulation efficiency. Similarly, through viral transduction (biological method), it is possible to load nucleic acids into exosomes stably, but the method suffers from the concerns about safety risks. Besides, the viral transduction approach is also laborious and time-consuming. In practical applications, it is still not satisfactory to use only one method to meet all the requirements. Hence, multiple approaches are sometimes used and new approaches for efficient and convenient loading are to be explored.

**TABLE 1:** Common approaches for cargo loading into exosomes.

Approaches	Advantages	Drawbacks	References
**Physical methods**			
Sonication	High loading efficiency, size reduction and removal of the naïve contents of exosome	Heat generation	Sancho-Albero et al., 2019; Jang et al., 2021
Electroporation	Powerful loading, ease in control	Aggregation of cargoes	Johnsen et al., 2016
Freeze-thaw	Mild process to load miRNA and proteins	Unspecific loading efficiency	Tran et al., 2019
Extrusion	Relative high loading efficiency, uniform size distribution of exosomes	Possible alteration the properties of exosome membranes	Fuhrmann et al., 2015; Haney et al., 2015
**Chemical methods**			
Saponin-assisted permeation	Incorporation of proteins directly, high encapsulation efficiency under right conditions	Hemolytic function	Fuhrmann et al., 2015; Sancho-Albero et al., 2019
Transfection	Convenience in the loading of nucleic acids	Toxicity to cells, difficulty in purification	Wahlgren et al., 2012; Wang Q.Z. et al., 2021
**Biological methods**			
Incubation	Simple operation, maintenance of the activity of cells/exosomes	Low encapsulation efficiency	Haney et al., 2015; Brossa et al., 2021
Viral transduction	Stably loading of nucleic acids into exosomes, possible enrichment of the exosome functions	Safety risks, laborious and time-consuming	Fu et al., 2020; Zhao X. et al., 2020

## Exosome Modification for Targeted Delivery to Specific Tissues or Cells

As a natural carrier of nucleic acids and proteins, exosomes have been considered to be crucial in intercellular communication and therefore extensively utilized as vectors for targeted delivery with biological molecules. Compared with the synthetic carriers such as liposomes or other nanoparticles, exosomes as drug delivery systems exhibit advantages of good biocompatibility, low immunogenicity and the capability to cross biological barriers such as the BBB.

**FIGURE 7:**
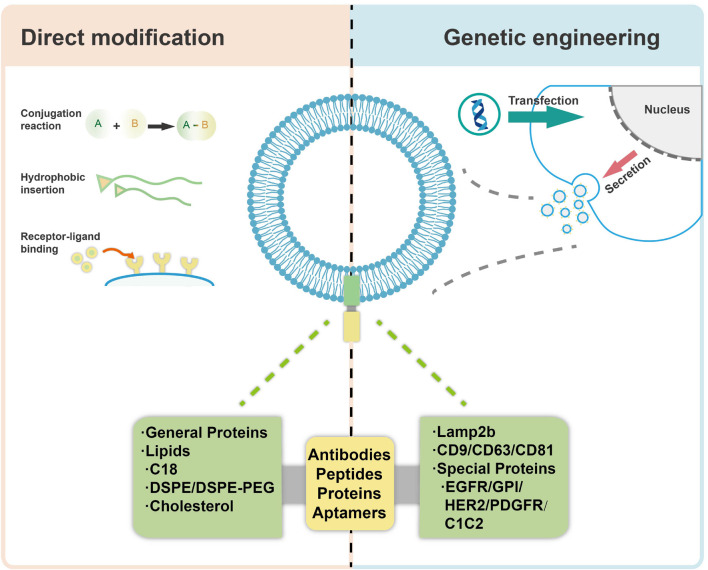
Modification of exosomes for targeted delivery *via* direct modification and genetic engineering.

Wiklander et al. (2015) isolated exosomes from three different murine cell sources and investigated their biodistribution in mice after systemic delivery. It was observed that the vesicles accumulated mainly in liver, spleen, gastrointestinal (GI) tract and lungs, and the different biodistribution patterns were related to their origins, the route of administration and the dose of vesicles injected. These data demonstrate the targeting capacity and potential of the exosomes, suggesting the cargoes in/on vesicles may be a critical factor leading to such effects. Moreover, modification of the exosomes should be a preferred way for specific targeting of tissues and/or cells in combination with their specific cell homotropic feature and origin (Morishita et al., 2015). In order to further utilize exosomes for specific targeting applications, there are mainly two approaches to design and manipulate membrane surface of exosomes, including a direct modification of exosomes (chemical modification) and indirect manipulation of exosome-secreting cells (genetic engineering) (Liang et al., 2021), as shown in Figure 7. Both of the two approaches could lead to higher drug accumulation in target cells, thereby reducing the off-target effects and consequently improving their efficacy. By employing the modification strategies, various bioactive ligands have been successfully incorporated with exosomes, which is shown in Table 2, with detailed descriptions on the specific methods used. As compared with genetic engineering method, which is complex and time consuming, direct modification of exosomes would be more reliable when the source cells for exosomes are not amenable to customized modification. However, direct modification of exosome surface proteins meets with problem of low specificity and low efficiency. Besides, it may also destruct crucial exosome components which may be functional for exosome-cell interactions and cargo delivery (Vader et al., 2016; Luan et al., 2017).

**TABLE 2:** Exosome modification approaches for targeted delivery.

Approaches	Bioactive ligands/transmembrane proteins	Methods	References
**Direct modification**			
Bioconjugation reaction	CP05	Directly bind to the second extracellular loop of CD63	Gao et al., 2018
	RGE-peptide	Cycloaddition reaction	Jia et al., 2018
	BMSC-specific aptamer	Schiff base reaction	Luo et al., 2019
Hydrophobic insertion	RGD peptide	Incubated in modified medium containing DSPE-PEG-RGD	Wang et al., 2017
**Genetic engineering**			
Non-specific proteins	Lamp2b	T7	Kim et al., 2020
		RAGE-binding peptide	Kim et al., 2021
		tLyp-1-lamp2b	Bai et al., 2020
		HuR	Li Z. et al., 2020
	The tetraspanin superfamily	CD9	Li et al., 2019
		The 2nd extracellular loop of CD63	Duong et al., 2019
		CD63-VEGFC fusion protein	Li B. et al., 2020
		CD63 truncations	Curley et al., 2020
Receptor membrane proteins	PDGFR	Human CD3 and HER2 receptors	Shi et al., 2020
	GPI	Anti-EGFR nanobodies	Kooijmans et al., 2016a
	HER2	DARPin G3	Molavipordanjani et al., 2020
	C1C2 domain of lactadherin	RGD-4C peptide	Tian et al., 2021
		Ag	Bliss et al., 2020

### Direct Modification of Exosomes

Direct modification of exosomes is carried out mainly through the conjugation reactions (including click chemistry), hydrophobic insertion, receptor-ligand binding and so on. Gao et al. (2018) have characterized a peptide, CP05, able to directly bind to the second extracellular loop of CD63 irrespective of the origin of exosomes, through conjugation reactions. Besides, systemic administration of exosomes loaded with CP05 (a peptide binding to the exosome marker CD63)-modified, dystrophin splice-correcting Phosphorodiamidate Morpholino Oligomer (EXOPMO) increased dystrophin protein 18-fold in the quadriceps of dystrophin-deficient mdx mice compared to CP05-PMO (Gao et al., 2018). Jia et al. (2018) loaded superparamagnetic iron oxide nanoparticles (SPIONs) and curcumin into the exosomes by electroporation, simultaneously conjugated RGE (a neuropilin-1-targeted peptide) to exosome membranes through a cycloaddition reaction with sulfonyl azide to obtain glioma-targeting exosomes, which exhibited considerable imaging and therapeutic functions. Furthermore, it was observed that the cellular uptake of exosomes can be specifically altered with a tethered AS1411 aptamer. After functionalization of exosomes with an immunomodulatory protein FasL, the exosomes demonstrated significant biological activity both *in vitro* and *in vivo* (Yerneni et al., 2019). Hydrophobic insertion approaches are easily achieved by direct co-incubation with cells or EVs. Recently, stromal cell (ST)-derived exosomes (STExos), conjugated with a bone marrow stromal cell (BMSC)-specific aptamer, successfully delivered STExos into BMSCs within the bone marrow. Intravenous injection of the STExos-Aptamer complex resulted in enhanced bone mass in ovariectomy mice, and accelerated bone healing in a femur fracture mouse model (Luo et al., 2019). For example, through a donor cell-assisted membrane modification strategy, the isolated exosomes exhibited increased targeting efficacy to blood vessels owing to the modified Arg-Gly-Asp (RGD) peptide on the exosome membrane. Meanwhile, the prepared exosomes presented a synergistic therapeutic effect and imaging capacity for angiogenesis both *in vitro* and *in vivo* (Wang et al., 2017).

### Genetic Engineering

Apart from the chemical modification of exosomal membranes, genetic engineering seems to be another way enabling efficient exosome modification. Modification of exosomes with targeting molecules can be achieved by genetic engineering of exosome producing cells, for example, through transfection with plasmid vectors. There are mainly two different categories of transmembrane proteins on the surface of the exosomes, non-specific proteins and receptor membrane proteins. The non-specific proteins mainly consist of lysosome-associated membrane protein 2b (Lamp2b) and the tetraspanin superfamily CD63/CD9/CD81 which are abundantly present on the surface of different exosomes.

Lamp2b is an extracellular surface protein with a signal peptide that is widely used for exosomal surface protein modification, especially for the delivery of RNA. It was proved that the N-terminus of LAMP-2B displayed on the surface of exosomes can be appended with targeting sequences. Kim et al. (2020) produced a T7 peptide-decorated exosome (T7-exo) by incorporation of T7 into the exosome membrane as a fusion protein of T7 and Lamp2b, which had a higher delivery efficiency to C6 glioblastoma cells than the unmodified exosomes and RVG-decorated exosomes *in vitro* and *in vivo*. In addition, the same group constituted RBP-linked exosomes (RBP-exo) by linking RBP (a RAGE-binding peptide) to an exosome membrane integral protein Lamp2b, followed by the loading with curcumin. As a result, the curcumin loaded RBP-exo exhibited a higher intracellular curcumin delivery efficacy than curcumin alone or curcumin loaded into unmodified exosomes (Kim et al., 2021). Besides, Bai et al. (2020) reported a novel tLyp-1 decorated exosome by transfection of HEK293T cells with tLyp-1-lamp2b. The results showed that the engineered tLyp-1 exosomes presented high targeting efficiency in lung cancer stem cells and the encapsulated siRNA was able to knock-down the target gene of cancer cells. In another case, the RNA-binding protein HuR was fused to the C-terminus of Lamp2b to form engineered exosomes, which decreased the abundance of RNA targets possibly *via* lysosome-mediated degradation, especially when the exosomes were acidified (Li Z. et al., 2020).

Additionally, considering their nature as transmembrane proteins and relatively abundant expression, the tetraspanin superfamily CD63/CD9/CD81 are often chosen for modification and protein fusion. Li et al. (2019) engineered exosomes for RNA loading by fusing CD9 with HuR, an RNA binding protein that interacts with miR-155 with a relatively high affinity, and revealed that the engineered exosomes could efficiently accumulate in the recipient cells, and recognize the endogenous targets. Duong et al. (2019) generated decoy exosomes by inserting TNFR1-EC at the 2nd extracellular loop of CD63. The engineered decoy exosomes specifically antagonize the activity of TNFα in cellular inflammation models (Duong et al., 2019). Li B. et al. (2020) have constructed engineered exosomes that highly bear a CD63-VEGFC fusion protein (CD63-VEGFC/exos), which promoted the proliferation, migration, and tube formation of lymphatic endothelial cells, and markedly improved lymphedema in a mouse model. Since the restrictive M-shaped topology of full-length CD63 may hinder specific applications of target protein in exosomes, Curley et al. (2020) conducted a series of CD63 truncations through sequential deletions of the transmembrane helix of CD63 and found the truncations retained robust membrane-anchoring and exosome-targeting activities.

On the other hand, receptor membrane proteins that are present on specific exosomes include epidermal growth factor receptor (EGFR), Glycosylphosphatidylinositol (GPI), HER2, platelet-derived growth factor receptor (PDGFR), the C1C2 domain of lactadherin, among many others.

Shi et al. (2020) utilized the human PDGFR transmembrane domain (TMD) as a fusion partner for genetic display of human CD3 and HER2 receptors’ functional monoclonal antibodies on the exosomal surface, and demonstrated the designed synthetic multivalent antibodies retargeted exosomes (SMART-Exos) exhibited highly potent and specific anti-tumor activity *in vitro* and *in vivo*. Kooijmans et al. (2016a) generated engineered exosomes with display of GPI-anchored anti-EGFR nanobodies, and showed the exosomes greatly improved exosome binding to tumor cells dependent of EGFR density. Moreover, the nanobody-displaying exosomes demonstrated significantly improved cell association to EGFR-expressing tumor cells under flow conditions (Kooijmans et al., 2016a). Engineered exosomes are also superior candidates for other clinical application such as tumor imaging. For example, ^99^mTc-radiolabeled exosomes that possessed DARPin G3 (as a ligand for HER2 receptor) not only displayed a higher affinity toward SKOV-3 cells (relatively high HER2 expression) in comparison to MCF-7, HT29, U87-MG, A549 cell lines (low HER2 expression), but were also able of tumor visualization in SKOV-3 tumor-bearing nude mice (Molavipordanjani et al., 2020).

As a member of the secreted extracellular matrix protein family, lactadherin is a small multifunctional glycoprotein that binds phosphatidylserine (PS)-enriched cell surfaces in a receptor-independent manner (Kamińska et al., 2018). Since the C1 and C2 domains (referred to as C1C2) readily self-associates onto the EV membrane, Tian et al. (2021) generated a recombinant fusion protein containing the arginine-glycine-aspartic acid (RGD)-4C peptide fused to C1C2—the PS-binding domains of lactadherin. It was verified the RGD-EV targeted the lesion region of the ischemic brain after intravenous administration, and resulted in a strong suppression of the inflammatory response (Tian et al., 2021). To overcome pre-existing immunity to commonly used human adenovirus serotype 5 (Ad5), Bliss et al. (2020) engineered targeting exosomes through fusion of Ag to the C1C2 domain of lactadherin, and revealed that exosomes induce substantially increasing Ag-specific humoral immunity post intramuscular and intranasal vaccination, improving the immunological potency of both ChAdOx1 and Ad5.

## Engineering for Prolonging Circulation

The targeted delivery of exosomes is highly restricted because of their rapid clearance from blood circulation after systemic administration. The short residence time in blood of 2–20 min post injection limits their applications for long and effective therapeutic action. Exosomes were initially postulated to be stable *in vivo*, as they are isolated from a variety of biological fluids with unique lipid, nucleic acid and protein compositions. However, recent investigations on exosome biodistribution demonstrated that intravenously injected exosomes are quickly sequestered by the liver, lung, and spleen (Sun et al., 2010; Takahashi et al., 2013; Lai et al., 2014).

Smyth et al. (2015) reported that the unmodified exosomes, traditional liposomes prepared with phosphatidylcholine/cholesterol, and liposomes formulated with the lipid extracted from exosomes exhibited a similar rapid clearance and low tumor accumulation after intravenous administration, suggesting that the naturally derived exosomes do not advance circulation retention or alter *in vivo* disposition. However, it was of significance that the exosome possessed a greater extent in tumor tissue through intratumoral injection when compared with the liposomes (Smyth et al., 2015). To perform pharmacokinetic studies, Takahashi et al. (2013) produced luciferase-presenting exosomes from B16BL6 cells, and found that the labeled exosomes disappeared from blood circulation rapidly with a half-life as short as 2 min after intravenous administration into mice. Although the biodistribution pattern may differ dependent on the cell sourcing, a significant portion of systemically injected exosomes were distributed to the liver, lung and spleen (Wiklander et al., 2015). Specifically, it was further proven that the clearance of exosomes from blood was mainly carried out by macrophage *via* phagocytosis (Feng et al., 2010; Imai et al., 2015), which is a receptor-independent event. The recognition of exosomal surface molecules, including phospholipids and proteins, is a critical mechanism in the phagocytosis of exosomes (Morishita et al., 2017). Recent investigations reported that deposition of phosphatidylserine, complement protein C3, and CD169 ligand on exosome surface may induce the macrophagic capture and uptake (Papp et al., 2008; Saunderson et al., 2014; Matsumoto et al., 2017).

Interestingly, an exosomal protein CD47 facilitates protection from phagocytosis, through interacting with its ligand signal regulatory protein α (SIRPα). CD47-SIRPα binding initiates a ‘don’t eat me’ signal that blocks the uptake by macrophages (Long and Beatty, 2013). Kamerkar et al. (2017) isolated CD47-bearing exosomes from normal fibroblast-like mesenchymal cells, and found that the presence of CD47 could suppress the clearance of exosome from the circulation. Although CD47-expressing exosomes have been identified from several cell types, such as fibroblasts, mesenchymal stem cells, and T cells (Kim et al., 2012; Kaur et al., 2014), bioengineering of augmented CD47 deposition on exosomes seems to be a promising approach to prolong the circulation time and alter the biodistribution.

Apart from natural molecules, the coating or incorporation of exosomes with synthetic materials can be another route to modulate the pharmacokinetics and biodistribution. Kooijmans et al. (2016b) modified EVs with PEG through a post-insertion mechanism, which prevented phagocytosis and resulted in a prolonged circulation time from 10 to 60 min. However, due to the shielding properties of PEG, cellular binding was compromised. Specifically, the conjugation of a targeting ligand to PEG was required to confer a selective biodistribution (Kooijmans et al., 2016b). With the control over the length and amount of conjugated synthetic polymer, Lathwal et al. (2021) applied atom transfer radical polymerization (ATRP) technique to decorate exosome surface using cholesterol-DNA tethers and various biocompatible monomers. The formed exosome-polymer hybrids achieved fourfold longer circulating retention with preserved bioactivity (Lathwal et al., 2021).

In summary, in order to improve the pharmacokinetics and biodistribution profiles, the physicochemical properties of exosomes can be bioengineered through coating with bioactive ligands or synthetic molecules. Such modulation is pivotal for employing exosome as cargo carriers for certain applications and particular forms of administration.

## Challenges and Potential Solutions

Compared to liposomes and other nanosized carriers, exosome-based delivery platform is superior in compatibility, stability, tissue penetration, targeting capacity and inherent bioactivity. However, it is worth to note that some limitations are restricting their translation into clinical practices for delivering therapeutic cargoes. Some of them are listed below.

First, the production yield of exosome is not high. The high cost of cell culture and time-consuming process for exosome release, isolation and purification severely limit the yield of cell derived exosomes. Breakthroughs in instruments and procedures have to be made to realize the large-scale production of exosomes or cargo-loaded exosomes. Recently, some strategies have already been employed to increase the production of exosomes through manipulating the gene expression and cell culture conditions (Jafari et al., 2020). Genetic manipulation of activating their biogenesis (e.g., overexpression of HSP20 (heat shock proteins 20), tetraspanin 6 or CD9) and inhibiting exosome recycling [e.g., negative regulation of PIKfyve (the phosphoinositide kinase, FYVE-type zinc finger)] can significantly enhance the secretion of exosomes (Hessvik et al., 2016; Wang et al., 2016; Guix et al., 2017; Schiller et al., 2018). Additionally, different control over the cell culture conditions can affect the exosome release from different cells. It was well demonstrated that hypoxia, inflammation stimulation and increased level of intracellular calcium concentration by treating with specific drugs could force the cells to produce more exosomes (King et al., 2012; Watson et al., 2012; Pusic et al., 2014; Messenger et al., 2018). Except for manipulations on gene expression and culture environment, three-dimensional culture system forming cellular spheroids could be another efficient strategy to scale up the production of exosome for clinical usage (Cha et al., 2018).

Second, a standardized control on exosome quality is lacking. Depending on the various statuses of different parent cells, exosomes are highly heterogenic in terms of the carrying nucleic acids and proteins. Undesired molecules may affect the function of exosomes and even arise the harmful or unwanted side-effects (Zhang et al., 2019). In order to ensure the safety of clinical usages of exosome, exercise strict control over the exosome source (e.g., species, cell types, and culture conditions) should be paid, and precise, sensitive and high-throughput analysis on their proteomics and genomics are desired. Furthermore, the isolated and purified exosomes are sometimes with a relatively broad size range, usually mixed with other EVs. Considering that size-dependent passive targeting may result in unpredictable biodistribution of exosomes, appropriate purification procedures should be included to separate exosome population in homogeneous size (Buschmann et al., 2021). Exosomes of narrow size distribution can be isolated and enriched through semi-continuous size exclusion chromatography technique (Moleirinho et al., 2019). Advancements on exosome production and evaluation will highly power exosomes as therapeutic tools in targeting delivery applications.

Third, exosome-based clinical trials remain a challenge. Credible and large-scale clinical trials are necessary and crucial to confirm and evaluate the therapeutic potentials before industrials activities (Chen et al., 2020). Currently, over 100 clinical trials on exosome-related investigations have been registered at Clinicaltrials.gov. For clinical trials of applying exosome as cargo carriers, MSCs and plant cells are commonly used as producer cells to load curcumin or genetic substances for the treatments of cancers and cerebrovascular disorders. A clinical study from Isfahan University of Medical Sciences on miR-124-loaded MSCs-derived exosomes for treating acute ischemic stroke is now at Phase II Trial (NCT03384433). In another study, M.D. Anderson Cancer Center is exploring the use of MSCs-derived exosomes loaded with KRAS G12D siRNA to treat metastatic pancreas cancer (NCT03608631).

## Conclusions and Perspectives

Targeted delivery using well-designed carriers enables the therapy of various diseases with high efficiency and reduced toxicity. With the advancements on biocompatibility, deep tissue penetration, multiple cargo loading capability, and surface modification tolerability, exosomes are of great interest and significance as natural carriers. In this review, we introduced exosomes as versatile carriers for targeted delivery. Diverse loading strategies facilitate the establishment of exosome-based carrier platforms to deliver various therapeutic molecules. However, breakthroughs in technologies and instruments should be solved to realize the large-scale production of purified and quality-controllable exosomes and drug-loaded exosomes. Engineering of exosomes with cell-specific recognition proteins or synthetic polymers facilitates targeted delivery and enhanced circulation time, without losing the unique features of exosomes. Current investigations on the pharmacokinetic profile and biodistribution of exosomes are mainly carried out in small animal models like mice. As the required steps toward a practical utility of the engineered exosomes, studies on large animals and then clinical trials should be conducted in the near future. On the other hand, the type of exosomes is one of the key concerns in the development of exosome-based delivery systems. In particular, for therapeutic application of drug/gene-encapsulating exosomes, the source of exosomes needs to be carefully considered. Different origins of exosomes confer them with different features and compositions. On the investigation and evaluation of the side effects and treatment efficacy of exosomes, the potential risk of inducing tumor growth by tumor cell-derived exosomes has to be taken into account. By introduction and discussion on the state-of-the-art designs and applications of advanced exosome-based targeted delivery system, this review aims to further encourage and inspire readers to develop new strategies for efficient, stable and safe targeted delivery systems in the future.

## Author Contributions

HC: writing – original draft, conceptualization, supervision, and funding acquisition. LW and XZ: writing – original draft. HS: supervision and writing – review and editing. HN: project administration, funding acquisition, and writing – review and editing. XP: supervision, project administration, and resources. YZ: conceptualization, supervision, project administration, funding acquisition, and writing – review and editing. All authors: read the revised manuscript and approved the submission.

## Conflict of Interest

The authors declare that the research was conducted in the absence of any commercial or financial relationships that could be construed as a potential conflict of interest.

## Publisher’s Note

All claims expressed in this article are solely those of the authors and do not necessarily represent those of their affiliated organizations, or those of the publisher, the editors and the reviewers. Any product that may be evaluated in this article, or claim that may be made by its manufacturer, is not guaranteed or endorsed by the publisher.
